# Mobile Phone Intervention to Reduce Youth Suicide in Rural Communities: Field Test

**DOI:** 10.2196/10425

**Published:** 2018-05-31

**Authors:** Anthony R Pisani, Peter A Wyman, Kunali Gurditta, Karen Schmeelk-Cone, Carolyn L Anderson, Emily Judd

**Affiliations:** ^1^ Department of Psychiatry University of Rochester School of Medicine and Dentistry Rochester, NY United States; ^2^ Department of Pediatrics University of Rochester School of Medicine and Dentistry Rochester, NY United States

**Keywords:** suicide prevention, school-based program, text messaging, school health services

## Abstract

**Background:**

Suicide is a leading cause of death among 10- to 19-year-olds in the United States, with 5% to 8% attempting suicide each year. Suicide risk rises significantly during early adolescence and is higher in rural and underserved communities. School-based universal prevention programs offer a promising way of reducing suicide by providing strategies for emotion regulation and encouraging help-seeking behaviors and youth-adult connectedness. However, such programs frequently run into difficulties in trying to engage a broad range of students. Text messaging is a dominant medium of communication among youths, and studies show both efficacy and uptake in text messaging interventions aimed at adolescents. Text-based interventions may, thus, offer a means for school-based universal prevention programs to engage adolescents who would otherwise be difficult to reach.

**Objective:**

We field tested Text4Strength, an automated, interactive text messaging intervention that seeks to reach a broad range of early adolescents in rural communities. Text4Strength extends Sources of Strength, a peer-led school suicide prevention program, by encouraging emotion regulation, help-seeking behaviors, and youth-adult connectedness in adolescents. The study tested the appeal and feasibility of Text4Strength and its potential to extend universal school-based suicide prevention.

**Methods:**

We field tested Text4Strength with 42 ninth-grade students. Over 9 weeks, students received 28 interactive message sequences across 9 categories (Sources of Strength introduction, positive friend, mentors, family support, healthy activities, generosity, spirituality, medical access, and emotion regulation strategies). The message sequences included games, requests for advice, questions about students’ own experiences, and peer testimonial videos. We measured baseline mental health characteristics, frequency of replies, completion of sequences and video viewing, appeal to students, and their perception of having benefited from the program.

**Results:**

Of the 42 participating students, 38 (91%) responded to at least one sequence and 22 (52%) responded to more than a third of the sequences. The proportion of students who completed multistep sequences they had started ranged from 35% (6/17) to 100% (3/3 to 28/28), with responses dropping off when more than 4 replies were needed. With the exception of spirituality and generosity, each of the content areas generated at least a moderate number of student replies from both boys and girls. Students with higher and lower levels of risk and distress interacted with the sequences at similar rates. Contrary to expectations, few students watched videos. Students viewed the intervention as useful—even those who rarely responded to messages. More than 70% found the texts useful (3 items, n range 29-34) and 90% (36) agreed the program should be repeated.

**Conclusions:**

Text4Strength offers a potentially engaging way to extend school-based interventions that promote protective factors for suicide. Text4Strength is ready to be revised, based on findings and student feedback from this field test, and rigorously tested for efficacy.

## Introduction

### Background

Suicide is the third-leading cause of death among 10- to 19-year-olds in the United States, and 5% to 8% of adolescents attempt suicide each year [1]. The risk for suicide and associated disorders (eg, depression, anxiety, and distress) rises during early adolescence [2]. The risk is higher in rural and underserved communities [3] where mental health services are less accessible or acceptable [4,5]. School-based universal prevention programs that address protective and risk factors across a population of students in early childhood [6,7] and adolescence [8-11] are emerging as viable ways to reduce youth suicide. Help-seeking behaviors, youth-adult connectedness, and strategies for the regulation of emotion are promising targets [11-13]. However, in the context of schoolwide prevention programs, reaching a diverse array of early adolescent students can be challenging, especially for students who are less engaged with school.

This study sought to overcome this challenge through a program of automated, interactive text messages (short message service [SMS]) that targeted help-seeking attitudes and norms, social coping resources, and emotion regulation skills in order to reinforce and extend school-based universal suicide prevention. Automated SMS text messaging interventions have proliferated in recent years, and studies have shown efficacy and uptake in adolescent populations. Interventions delivered through text messaging to youth enrolled in health behavior programs, such as diabetes control [14], HIV management [15], cancer posttreatment care [16], and substance abuse [17] have shown positive results. A pilot test of an automated SMS text-based intervention for adolescents who screened positive for depression and past-year violence in an emergency department was well received by patients and promising in terms of symptom improvement [18,19]. Few SMS texting interventions aimed at youth in the general population (outside of clinical contexts) have been tested for efficacy. However, studies of population-oriented programs such as Text4baby, SEXINFO, Hookup, and Smokefree Teen have shown that adolescents are often willing to adopt and use texting programs in large numbers [20-25].

To our knowledge, SMS text messaging has not yet been used successfully to extend a universal school-based intervention, nor to engage internal and social protective factors for suicide prevention. However, prior work suggested that individuals are often more willing to engage in social-emotional communication in text-based media than in face-to-face communication [26], perhaps because electronic media provide a layer of privacy that frees the individual to explore feelings and topics with which they might otherwise be uncomfortable. Most interventions delivered through text messaging to youth in clinical populations have not harnessed the potential of the medium for interactive communication, instead using one-way messaging or reminders to reinforce behaviors and direct the attention of participants toward core intervention concepts. One exception to this trend is the iDOVE program [18,19], which made limited use of interactivity, providing cognitive behavioral therapy coaching to adolescents with depressive symptoms who presented in a general emergency department. In response to automated daily prompts, participants could receive a single automated daily message or could initiate the receipt of a single additional support message if they were feeling sad, angry, or stressed.

In this paper, we report the results of field testing Text4Strength. Text4Strength was developed as a universal SMS text messaging intervention to strengthen protective factors for suicide prevention at the key transition point of high school entry [27]. This program extends Sources of Strength [11], an evidence-based peer network intervention in schools. Text4Strength seeks to promote healthy norms, attitudes, and behaviors by using a variety of novel interactive SMS text messaging sequences and by leveraging peer modeling and testimonials consistent with Sources of Strength.

### Text4Strength for Ninth Grade: Text Messaging Extension of Sources of Strength

#### Text4Strength and Sources of Strength

Sources of Strength is a program, certified by the US National Registry of Evidence-based Programs and Practices, that trains peer opinion leaders in messaging and social marketing activities that promote healthy social norms and communication with trusted adults, and help-seeking from adults for suicide concerns. In a randomized controlled trial (RCT) conducted in 18 schools, schoolwide help-seeking norms increased where Sources of Strength was implemented. Trained peer leaders were 4 times more likely to refer a suicidal friend to an adult [11]. An ongoing RCT [28] with 40 schools in rural and underserved communities is testing the impact of Sources of Strength on reducing self-reported suicide attempts. Text4Strength shares Sources of Strength’s core strategy of leveraging peer leaders’ creativity and positive modeling, and aims to extend both the reach and scope of Sources of Strength and other school-based interventions. Text messaging has the potential to reach students who are isolated, do not have close friendship ties, or are hesitant to participate in prevention activities in the school setting. In terms of scope, Text4Strength extends Sources of Strength concepts by teaching specific skills for the self-regulation of emotion in addition to reinforcing the core concepts of Sources of Strength.

#### Developmental Context

We field tested a version of Text4Strength that centers on the concerns of ninth graders in the United States as they transition to high school. As discussed below, research on early adolescence suggests that this period may be ideally suited for interventions focused on emotional skills, help-seeking skills, and supportive resources. High school provides opportunities for meeting new friends and mentors, as well as for academic growth and engagement in new activities, but it also presents a host of new stressors [29]. Furthermore, starting high school coincides with the pronounced increase in emotional and behavioral problems that occurs between the ages of 14 and 15 years [2,30]. On entering high school, ninth graders have to take on a greater responsibility than before for seeking help, rather than relying on adults to initiate help. Despite generally feeling that they receive greater support from peers than from adults, ninth graders’ perception of adult support is more strongly related than perceived peer support to their adjustment to the transition [31,32]. Incoming high schoolers also face greater academic pressures [30] and increased emphasis on dating relationships, as well as normal age-related developmental changes that result in mismatches between heightened emotional activation [31-33] and executive functions responsible for inhibitory control [34,35]. These mismatches contribute to adolescents’ greater susceptibility to suicide-related modeling [36].

#### Text4Strength Targets and Strategies

Text4Strength targets are (1) norms and attitudes toward help seeking, (2) connections with trusted adults, (3) connections with other social resources (eg, Sources of Strength), and (4) strategies for emotion self-regulation during the transition to high school (Table 1). Our previous work showed that high school students with suicide ideation were more likely to seek adult help if they held positive views toward seeking such help (eg, belief that friends and family would want them to ask for help), if they perceived adults at school as available and capable of helping suicidal students, and if they believed that the social resources in their lives (family support, positive friends, mentors, healthy activities, generosity, spirituality, mental health, and medical access) would help them get through tough times [12]. Positive help-seeking attitudes are also associated with both a lower risk for suicidal behavior [37] and increased help-seeking behavior among suicidal youth [12]. Numerous studies have demonstrated the importance of youth-adult connections in adolescent health [38-40] and for suicidal youth in particular [41-45]. Having trusted adults at school, at home, and in the community was associated with fewer suicide attempts in a large nonclinical sample of adolescents from an underserved community [13]. Maladaptive emotion self-regulation processes have been linked to key risk factors for suicide: depression [46-48], anxiety [49,50], antisocial behavior [51,52], and drug use [53]. Difficulties in various aspects of emotion regulation, including lack of emotional awareness [54], restrictive emotionality [48], and strategies for responding to and recovering from emotional upset [13,54-56], have been linked directly with suicide risk and attempts. The association between suicide attempts and difficulties in emotion regulation is moderated by having trusted adults [13].

Text4Strength focuses on three core emotion self-regulation skills [57]: (1) monitoring one’s own and others’ emotions, (2) reducing escalation of emotions, and (3) building relationships that aid in maintaining control and regaining equilibrium. These skills are critical in adolescent development, and the lack of such skills is associated with suicide risk [13].

### Text4Strength Text Messaging Strategies

Text4Strength introduces new strategies for engaging participants in order to ultimately promote positive health behavior changes. In addition to reinforcing concepts and norms related to help-seeking and making connections with trusted adults and other social resources, Text4Strength teaches emotion regulation strategies through interactions in a novel environment. This gives participants the opportunity for deeper personal reflection, particularly because of the option-rich nature of the intervention, which allows them to choose the extent to which they would like to interact with it.

Text4Strength targets the aforementioned constructs through a range of different interactive, automated SMS text message sequences. This approach is consistent with other qualitative research suggesting that adolescents prefer variety in text messaging [18]. Text messages included (1) brief videos and text-based testimonials generated by high school peer leaders and designed to draw participants in for further skill-building interactions, (2) direct questions inviting ninth-grade students to consider their own experiences and the support available for personal problems, (3) requests for advice for other students dealing with difficult situations, and (4) light-hearted games and activities such as Choose Your Own Adventure (students select among positive emotional strategies in response to a challenging social scenario) and Would You Rather (students choose from a series of alternatives that start off silly and humorous and become more serious, ie, different options for seeking adult help for a suicidal friend; Table 1). Positive peer modeling cuts across message types based on past research demonstrating the importance of peers in an array of prosocial and risky adolescent behavior [58,59], including suicidal behavior [36]. Almost all the Text4Strength messages offered participants choices in the content they would receive (eg, watching a video or answering a question), because individual risk, needs, and access to resources vary, necessitating universal interventions that are option-rich [13]. Peer leaders generated video- and text-based testimonials with the aid of an online coaching interface we developed previously called StoryPRIME. The program coached students through a step-by-step process of creating testimonials based on successful experiences of managing emotions and using coping resources. Our previous work showed that StoryPRIME produced relatable, relevant, and interesting text messages for ninth graders [60].

In this study, we examined the degree to which ninth-grade students (1) replied to text messages sent to them; (2) viewed peer-generated videos sent via links in SMS text messages; (3) judged the messages and videos as fun, appealing, and easy to use; and (4) judged the messages and videos as relevant and beneficial to them. We further explored how students with different levels of preexisting protective and risk factors interacted with and learned useful skills from text messages, and we examined the relationships between the degree of interaction with texts and students’ perceptions about the usefulness of the program.

**Table 1:** Text4Strength text message concepts and skills, and presentation formats.

Concepts and skills, and format		Example of one outgoing message in a sequence^a^
**Norms and attitudes toward help seeking**		
	Questions about individual experience and available support	“After a lot of stress, she took a risk and talked to her teacher, who became a mentor. Who could you go to at school if you were stressed?”
	Requesting advice	“Adults want to help, but sometimes they don’t realize you want to share something. If a student wants to speak [with a] teacher, how could they get their attention?”
	Games, challenges, and activities	“If a friend broke their leg in front of you but told you not to get help, would you... A) call 911 B) wait until they said it was OK to call C) order pizza” “What if your friend was really depressed but said not to get help? I’d... A) go [with] them to a counselor B) convince them it’s okay to get help C) say suck it up”
**Connections with trusted adults**		
	Peer video and text testimonials	“Sometimes family can be supportive during tough times. Here’s a video from Karen {VIDEO LINK} Who in your family can cheer you up??”
	Questions about individual experience and available support	“Sarah found adults in her life she could trust as mentors. Who is your most important mentor? (parent, family member, teacher, coach, neighbor, counselor, etc)”
	Requesting advice	“We want your advice…let’s say your friend got in a fight with his mom, has three papers due tomorrow, and has a soccer game tonight. Would you tell your friend to A) write his papers before the game B) skip the game C) ask his teacher or coach for help D) talk to his mom E) not sure”
	Games, challenges, and activities	“Hi, [high school] students say family, friends, and mentors can be there for you when you’re dealing with a tough time. Ready for a challenge? Tell someone in your family something you appreciate about them. Let us know how they responded.”
**Connections with other social resources**		
	Peer video and text testimonials	“Hey! Here’s a video from John from {School} {VIDEO LINK} sharing how he made friends in [high school]. Text FRIEND after you’re done watching.”
	Questions about individual experience and available support	“Have you felt like you were trying to find your place like John? Reply YES or NO”
	Requesting advice	“It can be hard to figure out who to be friends with in high school. What advice would you give somebody who felt like they are trying to find their place?”
	Games, challenges, and activities	“Generosity is a source of strength. Want a generosity challenge? Think of someone who might be a little down and how you could go out of your way to encourage/help them. See if you can do this every day this week! Good luck!”
**Strategies for emotion self-regulation**		
	Peer video and text testimonials	“Want to hear from Simone about how she dealt with handling a lot of things in [high school]? {VIDEO LINK}”
	Questions about individual experience and available support	“Hard feelings tell you many things. When they last a long time, it usually means you need other people involved. Could that be true here? YES NO or MAYBE”
	Games, challenges, and activities	“When you’re confused about how you’re feeling, you can do a gut check. There are three steps you can take. Want to try? YES or NO”

^a^Some examples may address more than one concept or skill.

## Methods

### Participants

A total of 175 ninth-grade students in 2 rural high schools attended informational meetings organized by their schools. Research staff provided information and parent permission packets inviting participation to the students in attendance. Students were paid a total of US $30 for their participation in surveys and feedback; payment was not contingent on reading or replying to messages. All study materials and recruitment procedures were approved by the University of Rochester Research Subjects Review Board, Rochester, NY, USA.

### Procedures

Participants completed baseline surveys before the intervention. They then received 28 interactive, automated text message sequences (16 with links to peer-leader videos) over 9 weeks (approximately twice per week). Each week was dedicated to 1 module: Sources of Strength introduction, positive friend, mentors, family support, healthy activities, generosity, spirituality, medical access, or emotion regulation strategies. The number of responses requested from students in a text message sequence ranged from 0 to 8. Student responses to texts, interactions with links, video views, and viewing times were logged automatically. Participants completed follow-up surveys within 2 weeks of completing the intervention. Baseline and follow-up surveys were administered by study staff either in homeroom or a class and took about 15 minutes to complete.

### Safety Protocols

None of the messages in Text4Strength invited students to share about suicide-related thoughts or risk-related behaviors; however, precautions were taken to account for the possibility that students could spontaneously send a message that caused concern. At enrollment, students and parents were informed of the safety protocols for the field test, and the limits of confidentiality were explained. Texts were not monitored in real time but were reviewed within 72 hours. If students replied to automated texts with any unexpected words, the texting system replied with school-specific information about how to get help during and after school hours, along with a reminder that their replies were not monitored in real time. When texts were reviewed within the 72-hour period, any messages that caused concern would be brought to the principal investigator, and further action would be taken as needed. In addition, at the end of each assessment, students received an on-screen message and a paper handout detailing counseling resources they could access if they were experiencing any emotional distress.

### Measures

#### Baseline Characteristics and Risk Factors

The baseline survey consisted of questions about demographics; frequency of mobile phone use; measures of depression, distress, anxiety, and coping and support and emotion regulation; expectations about ninth grade; and their social network, with questions meant to identify friends at school. For scales, we noted Cronbach alpha for this sample.

##### Frequency of Cell Phone Use

Students reported how often they used their mobile phones to send and receive texts, talk to people, post to social media, and play online games [61].

##### Depression, Distress, and Anxiety

To characterize our convenience sample, students completed a baseline measure of depression (Short Mood and Feelings Questionnaire [62,63], alpha=.96), psychological distress (Kessler Psychological Distress Scale [64,65], alpha=.94), and anxiety (Generalized Anxiety Disorder 7-item scale [66], alpha=.95).

##### Coping and Support and Emotion Regulation

To determine the status of students on target constructs at baseline and subsequently examine whether their status would be related to their degree of interaction or perceived usefulness, we measured the following at baseline: Sources of Strength coping [11,12] (alpha=.87), integration with peers [67] (alpha=.94), caring adults at school [68] (alpha=.86), adults to trust at school [13] (alpha=.95), and the Lack of Emotional Clarity and the Limited Access to Strategies for Emotion Regulation subscales of the Difficulties in Emotion Regulation Scale [69] (alpha=.84 and alpha=.89, respectively).

##### Social Network Out Nominations

Students were asked to name up to 7 close friends from a list of ninth-grade students at their school.

#### Interaction With Texts

Text messages were sent and tracked using a Web app developed at the University of Rochester for this program of research. Students provided their phone numbers at the end of the baseline surveys. We then entered these phone numbers into the texting system and used them to track texts sent to and received by each student. We calculated the number of replies for students and proportions of students responding to individual messages, along with total number of sequences completed. Total responses within each module (eg, introduction, positive friends, generosity) were also calculated. Sequences were considered complete once no more responses were requested for that particular sequence.

#### Interaction With Videos

Peer-leader testimonial videos were hosted on wistia.com (Wistia Inc, Cambridge, MA, USA). Outgoing links from the text messages to wistia.com were tracked via link management site bit.ly (Bitly, Inc, New York, NY, USA) using unique URLs for each student. The video hosting platform tracked both the number of views and the percentage of each video watched by a given viewer.

#### Appeal of Text4Strength

We measured the appeal of Text4Strength using questions designed specifically for this study about the appeal of the text content, whether students shared the content with family or friends, and whether they felt comfortable texting (eg, “I read messages even when I didn’t reply back;” “It was fun to reply to messages and see what responses I’d get.”). Students were also asked questions based on the System Usability Scale [70-72], examining the ease and appeal of using the texting system itself, as well as about problems they encountered in receiving texts and viewing videos. Those who had seen at least one video were asked about having time to watch videos, the appeal of videos, and whether they were useful (eg, “I could relate to the peer leaders’ stories;” “I preferred videos made by peer leaders who go to our school.”). All questions were rated on a 5-point scale ranging from 1 (“strongly disagree”) to 5 (“strongly agree”).

#### Perceived Benefit

We asked students whether the texts were relevant or helpful to ninth graders and whether the students recommended that ninth graders should receive these messages in the following school year (eg, “The texts helped me in my transition to high school;” “Next year the 9th graders at my school should get these texts.”).

#### Student Feedback

Students responded to 6 open-ended items inviting them to state what they found most useful and to give their ideas for improving the program. The items were “What was most useful to you as a 9th grader?,” “What would make the program more useful for 9th graders?,” “What would make the texts more fun or enjoyable?,” “The video I remember most is...,” and “What would make the video more interesting and useful?”

### Analyses

We examined engagement with texts by coding valid responses to all delivered text messages and creating variables for total number of responses to each sequence and overall number of responses. By examining response patterns, we determined whether each participant had completed each sequence and created a sum of sequences completed. Similarly, we were able to determine the number of views for all videos and the amount of video that had been viewed. For both types of engagement, we examined whether the proportions of students replying differed by sex and school using independent-samples *t* test and, for text engagement, by depression, distress, anxiety, and coping and support and emotion regulation using correlations.

We examined the relationship between baseline characteristics (depression, distress, and anxiety; coping and support and emotion regulation) and survey responses to items on appeal and usefulness of the text messages using correlations for continuous variables and *t* tests or analysis of variance for grouped variables (ie, sex, depression cutoff, and psychological distress groups). We also examined free responses to the students’ suggestions. All statistical analyses were conducted in IBM SPSS v22 (IBM Corporation).

## Results

### Baseline Characteristics and Risk Factors

Of the 175 ninth-grade students who attended the information meetings, 58 (33.1%) returned completed parental permission packets and 53 (30.3%) participated in the initial survey held a few weeks later. Of these, 42 (79%, 30 female, 12 male) had mobile phones and could participate in receiving text messages and interacting with them; 2 students did not complete the follow-up survey. A total of 39 students (93%) identified as white, 1 as American Indian, 1 as Asian, and 1 as biracial (white and Asian); 4 students (10%) were 13 years old or younger, 35 (83%) were 14 years old, and 3 (7%) were 15 years or older.

Of the 53 students who completed the baseline measure, 37 (86%) reported sending and receiving text messages every day. Most students reported very few depressive symptoms (mean total depression score 5.28 out of 39), but about a quarter of the sample (15 students, 28% of total) reported symptoms above the threshold recommended by Angold et al [62] for identifying those who are likely to have clinically significant depression. Students generally reported very few symptoms of psychological distress (mean 1.75, SD 0.89; possible range 1-5). Students reported very few symptoms of anxiety (mean 0.59, SD 0.86; possible range 0-3). Students reported high use of Sources of Strength coping resources (mean 3.43, SD 0.51; possible range 1-4). Students reported having high levels of support from peers (mean 3.19, SD 0.83), caring adults at school (mean 3.21, SD 0.75), trusted adults at school (mean 3.28, SD 0.70), and other adults (mean 3.35, SD 0.69), where the possible range was from 1 to 4. Students reported sometimes feeling clear about their emotions (mean 2.24, SD 1.00; possible range 1-5) and sometimes having access to emotion regulation strategies (mean 1.82, SD 0.88; possible range 1-5). A total of 51 students (96%) named at least 1 friend, 17 (32%) named between 1 and 6 friends, and 34 (64%) named 7 friends.

### Interaction With Texts

Of the 42 participating students, 38 (91%) responded to 1 or more messages; 22 (52%) replied to at least one-third of the sequences, 13 (31%) replied to at least one-half of the sequences, and 8 (19%) replied to two-thirds or more. The number of students who completed multistep sequences they had started ranged from 35% (6/17) to 100% (3/3 to 28/28). Students who replied to the first text in a sequence completed the whole sequence on average 58% of the time (average of percentage completion).

**Figure 1:**
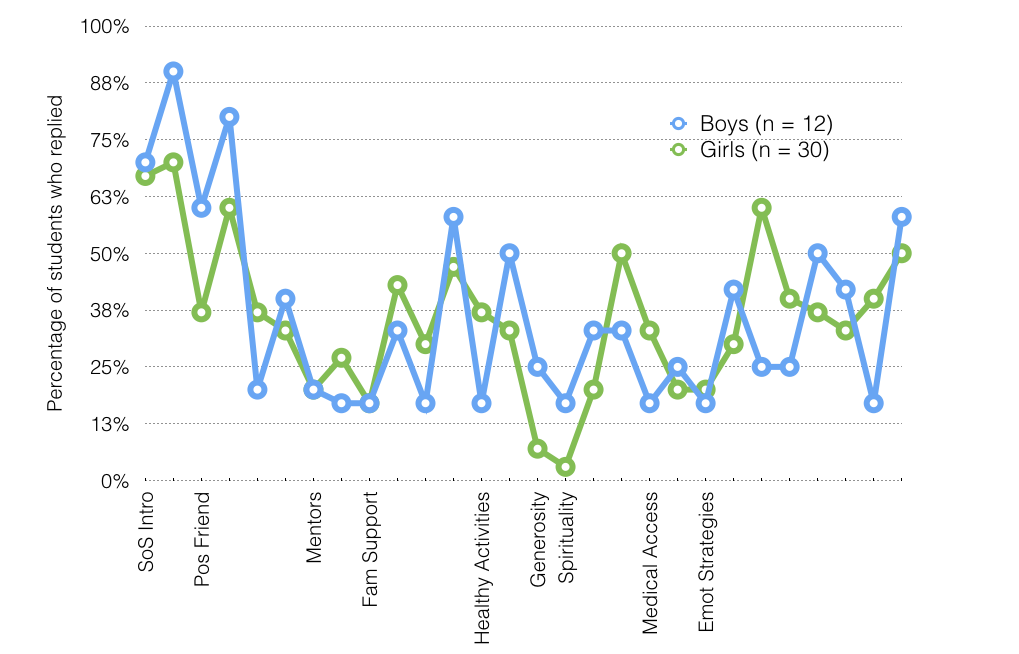
Percentage of girls and boys who replied to each of 28 sequences over 9 weeks of intervention. Emot Strategies: emotion regulation strategies; Fam: family; Pos: positive; SoS Intro: Sources of Strength introduction.

**Figure 2:**
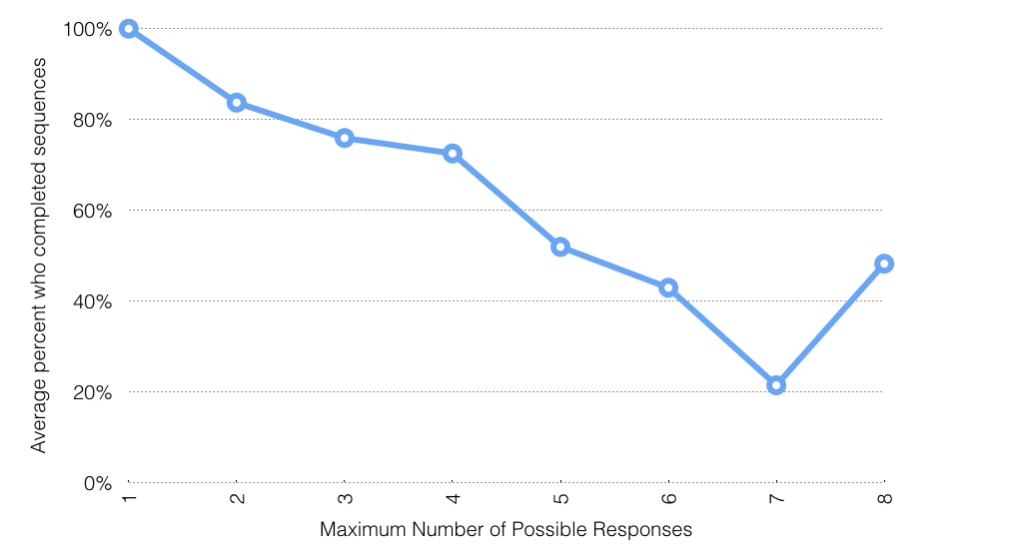
Average percentage of students who completed sequences by length of sequence (n=41).

The proportion of students replying to messages was not related to their sex, except in the generosity and spirituality modules, or to their school, previous reported use of texting, depression (*t* test), psychological distress (using analysis of variance for distress groups), anxiety, or coping and support or emotion regulation scales (correlation). Students with higher depressive symptoms (above the suggested cutoff, n=14), high psychological distress (above the suggested cutoff, n=5), or high anxiety (upper quartile, n=13) replied to a number of texts similar to that of students with few symptoms of depression, distress, or anxiety (8.80 vs 9.00 responses). We did not test the significance of these analyses because of the small cell sizes. As Figure 1 shows, levels of interaction across the 28 message sequences varied significantly, with the proportion of students replying ranging from 7% (3/42) to 70% (28/40), but proportions for each sequence did not, with 2 exceptions, differ significantly by sex (boys: mean 36.01%, SD 20.04%; girls: mean 32.98%, SD 16.41%; *t*_27_=1.161, *P*=.26). Similar numbers of students responded to most categories of sequences, with the exception of the sequences in the spirituality and generosity modules. The overall number replying to the single message sequence in each of these 2 modules was significantly lower than for any other module. While the number of students replying was within the normal variation for boys, very few girls replied to these 2 sequences. As Figure 2 shows, sequences longer than 4 conversational turns were completed by significantly fewer students.

### Interactions With Videos

Very few students followed links to view videos; however, when they did, they usually watched all the way through. As Table 2 shows, when offered a link to a video, between 2 and 22 (5%-52%) students (mean of 20%) watched it. Students who watched viewed an average of 86% of the video they watched (range 58%-100%). Students watched videos all the way through 69% of the times they started them. A total of 14 students (34%) reported that they were not able to view videos on their phones, 7 (50%) because they did not have internet access, 5 (36%) because their phone was slow, and 1 (7%) because the links did not work. Engagement with videos did not vary by sex or school. Students who showed signs of risk on their baseline surveys (high depressive symptoms or psychological distress or anxiety, as defined above), on average, viewed fewer videos than other students (less likely to access videos and watched fewer: 2.36 vs 3.26 videos).

**Table 2:** Students’ engagement with videos.

Video title	Students who viewed the video, n (%)	Average % of video viewed by those who watched
Finding My Place	22 (55)^a^	92.29
Everyone’s Really Cool	12 (30)	85
You Can Love Food and Still Fit In	9 (23)^a^	96.58
You Don’t Really Have to Impress Anyone	6 (15)^a^	60.5
Shakespeare Struggle	3 (8)	100
If I Knew Then What I Know Now	10 (24)^a^	77.75
Time Time Time	2 (5)	100
Do Things That Make You Happy	11 (26)	80.35
Find People Who Make You Laugh and Happy	2 (5)	58
Get Your Emotions Out There	22 (55)	73.62
Try New Things	5 (12)	90.13
Don’t Be Afraid to Ask for Help	3 (7)	97.75
Talk About It	9 (21)	84.42
Getting Teachers’ Attention Puppet Show	6 (14)	100
Bundled Up with Schoolwork and Activities	4 (10)	89.75

^a^Video appears later in message sequence (not in opening line).

### Appeal of Text4Strength

Student ratings of the text messages indicated that they found the texts to be fun, appealing, and easy to use. Most students felt that the frequency of texts (about twice per week) was right (n=3, 7% too few, n=5, 12% too many, n=33, 81% about right). As Table 3 shows, almost all students (n=40, 98%) read the messages even when they did not reply. A total of 17 (43%) students had talked to either friends or their parents or family about the texts. Among those who watched any videos, 70% (19) of the students liked the videos and only 7% (n=2) thought that the program would be better without videos. Students enjoyed hearing personal stories from upperclassmen (n=25, 93%).

Most students found the system easy to use, and only a few (7-8, 17%-19%) found it complicated or thought it took a while to figure out what to do. A total of 25 students (61%) liked the response they received when they replied with advice or experiences, while 14 students (34%) felt the system needed improvement. The Text4Strength system performed reliably. We had only 1 substantive technical glitch—students from 1 of the schools received multiple, repeat messages on a single day. Our new notification system functioned properly by alerting us to the problem based on unexpected student replies. We fixed the problem and apologized to students.

There were no adverse safety concerns. However, we encountered one situation in which an ambiguous reply text from a student required clarification to be sure she was not expressing distress. Following our approved safety protocol, we contacted the student, notified school partners, and were quickly reassured about her safety. In specific survey questions assessing possible negative effects, 2 students said 1 or 2 messages made them feel sad—in both cases, sadness was in response to hearing about hard situations faced by other students (eg, a parent leaving for military deployment) and feeling empathy for them.

Students participated vigorously in free-response suggestions and feedback. To make texts more enjoyable, 8 students suggested adding more or different games to message sequences. In addition, 6 students requested inserting more humor, such as jokes or cartoons, with more serious message content, and 6 students suggested using a more personal touch in text content and tone. Also, 1 student asked if the program could “make it like it was more of a best friend instead of a program.” This level of personalization came up in different ways throughout the course of the final survey. Students “liked when it asked me questions like how my day was.”

### Perceived Benefit

**Figure 3:**
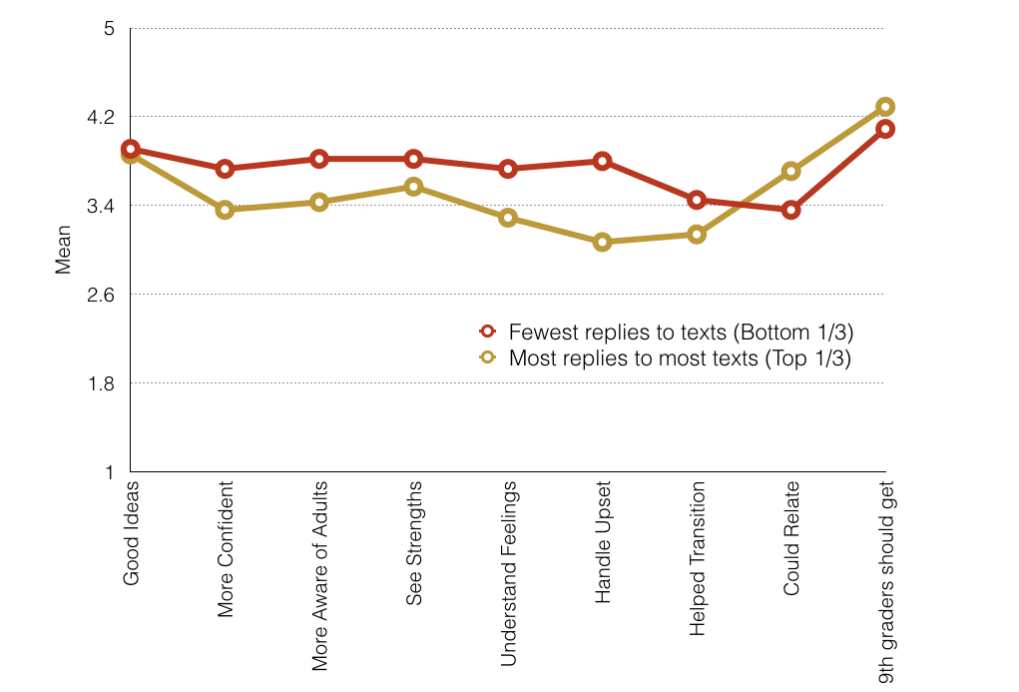
Equally "useful" for least- and most-engaged students.

Students found the text messages appealing and useful to the ninth-grade experience (Table 3), regardless of whether they replied frequently or not. Over 70% of students thought the texts gave them good ideas (n=34, 86%), helped them feel more confident with high school challenges (n=31, 78%), and made them more aware of adults they could access (n=29, 73%). In addition, 58% (n=23) felt the texts helped them with their transition to high school and 90% (n=36) agreed that ninth graders should receive the texts next school year. Students indicated that they had learned new ways to handle emotionally upsetting situations and that they understood their feelings and strengths better. As Figure 3 shows, the top third of students who replied to the most texts and the bottom third of students who replied to the fewest texts found the texts equally useful (*P*=.52 for difference in mean of items, *P* range .10-.90 for items).

**Table 3:** Appeal and usefulness of Text4Strength messages and videos.

Measure		Response, mean (SD)	Response, n (%)				
			Strongly disagree	Disagree	Neither	Agree	Strongly agree
**Message appeal (n=41)**							
	I enjoyed getting text messages from Sources of Strength	4.05 (0.545)	0	0	5 (12)	29 (71)	7 (17)
	I read messages even when I didn’t reply back	4.41 (0.55)	0	0	1 (2)	22 (54)	18 (44)
	It was fun to reply to messages and see what response I’d get	3.93 (0.685)	0	1 (2)	8 (20)	25 (61)	7 (17)
	I talked with my friends about texts I received	2.83 (1.05)	4 (10)	13 (32)	11 (27)	12 (29)	1 (2)
	I talked with my parents/family about texts I received	3.12 (1.19)	4 (10)	9 (22)	11 (27)	12 (29)	5 (12)
	I got bored with the messages after a while	2.73 (0.975)	2 (5)	19 (46)	9 (22)	10 (24)	1 (2)
	I didn’t take the messages seriously	2.02 (0.85)	12 (29)	18 (44 )	9 (22)	2 (5)	0
	I liked being able to share my own experiences and advice	3.85 (0.76)	0	2 (5)	9 (22)	23 (56)	7 (17)
	I felt comfortable texting personal things when asked	3.51 (0.90)	0	7 (17)	10 (24)	20 (49)	4 (10)
**Texting system usability (n=41)**							
	I liked texting back and forth with the Sources of Strength texting system	3.78 (0.725)	0	2 (5%)	10 (24)	24 (59)	5 (12)
	I found texting back and forth with the texting computer too complicated	2.71 (0.955)	4 (10)	13 (32)	16 (39)	7 (17)	1 (2)
	It took me a while to understand what I was supposed to do when I got a text from Sources of Strength	2.51 (1.075)	7 (17)	15 (37)	12 (29)	5 (12)	2 (5)
	It was easy for me to use keywords or letters to reply to texts	3.85 (0.76)	0	3 (7)	6 (15)	26 (63)	6 (15)
	When I replied with advice or experiences, I liked the response I got back	3.61 (0.89)	1 (2)	3 (7)	12 (29)	20 (49)	5 (12)
	The back and forth texting system would need to be improved before students will enjoy using it	3.07 (1.08)	3 (7)	9 (22)	15 (37)	10 (24)	4 (10)
**Message usefulness for ninth graders (n=40)**							
	The texts gave good ideas for 9th graders to follow	4.00 (0.72)	0	2 (5)	4 (10)	26 (65)	8 (20)
	The texts helped me feel more confident to face challenges in high school	3.70 (0.88)	2 (5)	1 (3)	8 (20)	25 (63)	4 (10)
	The texts made me more aware of adults I could talk to	3.75 (0.78)	1 (3)	2 (5)	6 (15)	28 (70)	3 (8)
	The texts helped me in my transition to high school	3.50 (1.01)	2 (5)	4 (10)	11 (28)	18 (45)	5 (13)
	As a 9th grader, I could relate to the situations described in the texts	3.78 (0.86)	1 (3)	3 (8)	5 (13)	26 (65)	5 (13)
	Next year, the 9th graders at my school should get these texts	4.30 (0.65)	0	0	4 (10)	20 (50)	16 (40)
**Strengths and emotions (n=40)**							
	The texts helped me see my own strengths	3.83 (0.90)	2 (5)	1 (3)	5 (13)	26 (65)	6 (15)
	The texts helped me understand my own feelings better	3.68 (0.92)	2 (5)	2 (5)	7 (18)	25 (63)	4 (10)
	I learned new ways to handle emotionally upsetting situations	3.56 (1.05)	3 (8)	3 (8)	6 (15)	23 (59)	4 (10)
**Video appeal (n=27)**							
	The videos I watched were interesting	3.74 (0.76)	0	2 (7)	6 (22)	16 (59)	3 (11)
	I could relate to the peer leaders’ stories	3.73 (0.78)	0	2 (8)	6 (23)	15 (58)	3 (12)
	My friends and I handle challenges similar to those described	3.59 (0.75)	0	2 (7)	9 (33)	14 (52)	2 (7)
	I recognized the students in the videos	4.04 (0.71)	0	1 (4)	3 (11)	17 (63)	6 (22)
	I preferred videos made by peer leaders who go to our school	4.11 (0.85)	0	2 (7)	2 (7)	14 (52)	9 (33)
	I thought peer leaders were being really honest in the videos	4.07 (0.73)	0	1 (4 )	3 (11)	16 (59)	7 (26)
	I didn’t have time to watch videos	2.44 (1.09)	5 (19 )	11 (41)	6 (22)	4 (15)	1 (4)
	The program would be better without videos	2.00 (0.96)	10 (37)	9 (33)	6 (22)	2 (7)	0
	The videos gave me a good impression of Sources of Strength	3.93 (0.88)	0	1 (4)	4 (15)	18 (67)	4 (15)
	I liked hearing personal stories from upperclassmen	4.00 (0.555)	0	1 (4)	1 (4)	22 (82 )	3 (11)
	Peer leaders didn’t talk about things I’m going through	3.12 (0.95)	1 (4)	6 (23)	9 (35)	9 (35)	1 (4)

### Student Feedback

In free-response feedback, students made suggestions for increasing the intervention’s usefulness for ninth graders in the future. One student said “I think that each student’s survey should be taken into consideration [so they] receive personalized messages [they] could relate to.” Along with suggestions for improvement, there were some complaints. When asked what would make the program more useful, 1 student wrote “nothing really, the texts didn’t really help all that much.” Several students asked that the program no longer include its automatic response at the end of each sequence: “don’t say ‘thank you for your message’.” Overall, however, the responses received in the final surveys were positive and constructive in nature, and students seemed genuinely invested in helping create a better program for future student participants. A sampling of common responses from the final survey follows.

In reply to the question “What was most useful to you as a 9th grader?”:

Knowing that some other people felt the same way or were going through the same things i could and could relate.

The most useful thing as a 9th grade related to the text was the ideas and advice about how to handle situations that have happened to me or could happen because it prepared me for the situations.

The reassurance that I COULD get through each and every day, Although some days felt as though there were more struggles that others, at the end of the day I was able to look at situations and events more-so optimistically and from various perspectives.

In reply to the question “What would make the texts more fun or enjoyable?”:

If there were more games that resulted in real life scenarios.

Getting replies from people instead of a computer.

Doing little cartoon videos about the things and problems kids are facing, so that if they are sad or down it would/could make them laugh.

In reply to the question “What would make the program more useful to 9th graders?”:

...maybe more meetings and activities, instead of just texting...because its easier to talk about your feelings with someone, instead of text them so someone.

The program would be more useful if there was more stories about situations other students were in that they could tell future 9th graders.

...make messages more interactive.

In reply to the question “What suggestions do you have for improving students’ experience of texting back and forth with the computer?”:

To make it for the kids who need it the most or seem like they need advice.

Maybe the computer can turn the question into a poem once in a while, but not a big poem, a small one.

...don’t have the same automated response every time a students texts and is not responding to a question.

In reply to the question “The video I remember most is:”

One of the videos I remember the most is one where I recognized the student and talked about how she got through a rough situation.

A girl’s dad going to Afghanistan.

The girl with from my school talking about your problems to others.

In reply to the question “What would make the videos more interesting and useful?”:

I think the videos would be more interesting if the videos were all from students I recognize.

...if they were based off of issues that have really happened to them.

If they were more fun.

## Discussion

### Principal Findings

Students who participated in this field test found Text4Strength to be appealing and useful, indicating that the text messaging program has potential for extending school-based interventions that promote protective factors for suicide. Ninth-grade students across a spectrum of risk and distress engaged with diverse text messages aimed at building coping resources and encouraging communication with trusted adults. Similar levels of interaction between those with higher and lower levels of risk and distress suggest that both struggling and relatively healthy adolescents may be willing to engage on their phones with material aimed at building protective factors if it is presented in the form of positive, fun, and appealing text messages. This is especially notable given that the sample included few highly distressed students. Most importantly, students reported that they gained awareness of their own feelings and learned new ways to handle upsetting situations.

Students found the texting system easy to use and there were no technical difficulties. Levels of student engagement with the text sequences were promising even though a substantial portion of students chose to reply to only 1 or 2 messages. Almost all students replied to at least 1 message and read messages even when they didn’t reply. A third of students engaged actively, replying to at least one-half of the sequences, and most students completed the text sequences and videos they started. Significantly, students found texts useful whether or not they replied often and recommended that the program be offered to students in the future, suggesting that they saw the texts as invitations to interact when an interest or need arose. Understanding this attitude can help us set realistic expectations about participation and adjust content so that modest use results in sufficient exposure.

To our knowledge, our study is the first to use text messaging to extend a universal school-based intervention specifically for ninth graders. As they transition to high school, ninth graders face several new and unique challenges and are in a period ideally suited for an intervention that engages internal and social protective factors for suicide prevention [29]. Text4Strength targets messages to a broad population, going beyond the clinical populations on which previous research has focused [14-17]. Even among text messaging interventions aimed at nonclinical populations [20-25], Text4Strength is the only one with a high degree of two-way interactivity and a variety of message formats [73].

Lessons from this field test and feedback from students can inform the next iteration of this intervention and the development of future text messaging interventions. First, the number of students replying to messages in most modules was fairly consistent, but almost no girls replied to messages we sent about spirituality and generosity. Future implementations of Text4Strength will need to improve on the content of these two categories if they are to be retained. Second, based on the observation that the number of students replying dropped significantly after the fourth message in a sequence, future iterations should either limit sequence length or experiment with new ways to hook students’ interest midsequence. Third, few students watched the videos. Students reported being limited by time, location, and bandwidth or capability. Text messaging is an on-the-go medium but videos require stopping to watch. Future universal texting interventions should carefully weigh the cost and value of producing videos. Fourth, Text4Strength leveraged peer voices through text- and video-based testimonials, but it was not tightly integrated with schoolwide messaging activities. More explicit links with school-based prevention activities might increase engagement with text message and video content. Fifth, consistent with other studies [18,74], in this study, students reported wanting more humor and personalization.

### Limitations

Several limitations should be noted. First, the primary limitation is that this was a field test with a small convenience sample of ninth-grade students in 2 rural high schools. It was designed specifically to inform a future pilot RCT. Despite the small sample, participants did not vary greatly from the populations of their respective schools on broad demographic factors or on Cronbach alphas and means for scales. The one exception was in the sex of participants; proportionally more girls than boys participated in the field test. Second, because of the novelty of the intervention, we lack data from the literature with which to benchmark the proportion of students interacting with texts and videos and to judge “successful” engagement. We hope that the proportions of students we documented replying to texts and viewing videos will provide that context for future research. Third, we tested only one style of video, namely peer testimonials. Thus, we cannot conclude definitively that video links in text messages could never work. Students who watched videos liked them and usually watched them all the way through, so perhaps a better description or “hook” for the videos could have resulted in more views.

### Conclusion

In this field test, Text4Strength was appealing and useful to students and technically feasible. Text4Strength offers a potential way to extend school-based interventions aimed at promoting protective factors for suicide. Text4Strength is ready to be revised, based on findings and student feedback from this field test, and rigorously tested for efficacy, including its effect on suicide-related outcomes. The most important revisions will be (1) making text messages more humorous and fun (including more games), (2) personalizing messages with students’ names and other tailoring, (3) reducing reliance on videos, and (4) integrating messages into concurrent school-based prevention activities of Sources of Strength. In addition, an efficacy trial of this intervention should include testing outcomes for higher-risk students, as well as the broader population. These changes are expected to increase overall engagement, leading to better retention and application of concepts and skills.

## Abbreviations

RCT: randomized controlled trial
SMS: short message service
